# Liver Fibrosis Is Associated With Hemorrhagic Transformation in Patients With Acute Ischemic Stroke

**DOI:** 10.3389/fneur.2020.00867

**Published:** 2020-09-10

**Authors:** Cheng-Xiang Yuan, Yi-Ting Ruan, Ya-Ying Zeng, Hao-Ran Cheng, Qian-Qian Cheng, Yun-Bin Chen, Wei-Lei He, Gui-Qian Huang, Jin-Cai He

**Affiliations:** ^1^Department of Neurology, The First Affiliated Hospital of Wenzhou Medical University, Wenzhou, China; ^2^School of Mental Health, Wenzhou Medical University, Wenzhou, China

**Keywords:** liver fibrosis, acute ischemic stroke, hemorrhagic transformation, risk factors, liver disease

## Abstract

**Background:** Hemorrhagic transformation (HT) is a frequent, often asymptomatic event that occurs after acute ischemic stroke (AIS). Liver fibrosis, usually subclinical, is common and crucial in the development of liver disease. We aimed to investigate the association between liver fibrosis and HT in patients with AIS.

**Methods:** We performed a single-center and retrospective study. A total of 185 consecutive participants with HT and 199 age- and sex-matched stroke patients without HT were enrolled in this study. We calculated one validated fibrosis index—Fibrosis-4 (FIB-4) score—to assess the extent of liver fibrosis. HT was detected by routine CT or MRI and was radiologically classified as hemorrhagic infarction type 1 or 2 or parenchymal hematoma type 1 or 2. HT was also classified into asymptomatic or symptomatic. We used logistic regression models adjusted for previously established risk factors to assess the risks for HT.

**Results:** The median FIB-4 score was significantly higher among patients who developed HT than among those without HT, whereas standard hepatic assays were largely normal. Patients were assigned to groups of high FIB-4 score and low FIB-4 score based on the optimal cutoff value. Compared with the subjects in the low-FIB-4-score group, incidence of HT for the high-FIB-4-score group was significantly higher. After adjustment for potential confounders, the patients with high FIB-4 score had 3.461-fold risk of HT in AIS compared to the patients with low FIB-4 score [odds ratio, 3.461 (95% CI, 1.404–8.531)].

**Conclusion:** Liver fibrosis, measured by FIB-4 score, was independently associated with the risk of HT in AIS patients.

## Introduction

Liver diseases could increase the risk of cardiovascular disease and lead to worse hospital discharge disposition and higher in-hospital mortality after stroke (1–3). Non-alcoholic fatty liver disease (NAFLD) is the most common cause of liver dysfunction, affecting about 25% of the adult population globally, which spans pathologies ranging from simple fatty liver (steatosis) to necroinflammatory non-alcoholic steatohepatitis (NASH), including scarring (fibrosis) and the formation of nodules surrounded by fibrotic bands (cirrhosis) (4, 5). A previous study has demonstrated that liver cirrhosis is associated with an increased risk of stroke, particularly hemorrhagic stroke (6). In addition, a recent study showed liver fibrosis, not simple steatosis, is a strong predictor of long-term mortality in the ischemic stroke population (7). Although these studies highlight a possible relevance between advanced liver diseases and poor stroke outcomes, it is still unsure if these findings can also apply to subclinical liver disease.

Liver fibrosis, a histological precursor to cirrhosis, is an often clinically (4) chronic liver disease and is preceded and promoted by an inflammatory process in conjunction with the accumulation of extracellular matrix in the liver (8). Studies previously have indicated an unexpectedly high prevalence of liver fibrosis in up to 9% individuals without known liver disease (9, 10). Furthermore, the presence and severity of liver fibrosis can predict cardiovascular mortality in patients with chronic liver disease as well as the risk of ischemic stroke (11, 12) and are associated with the outcomes after primary intracerebral hemorrhage (ICH) according to previous studies (13).

Hemorrhagic transformation (HT) is a common and severe complication that patients may develop in acute ischemic stroke (AIS) (14–16). And it is a major cause of early mortality and disability, which is potentially linked with clinical deterioration and poor outcomes (17–19). Previously identified risk factors for HT in ischemic stroke patients include increasing age (20), higher systolic blood pressure (SBP) (21), atrial fibrillation (22), thrombolysis (23), and symptom severity (24). In fact, several studies have found that liver fibrosis is associated with cerebral microbleeds and admission hematoma volume, hematoma expansion, and mortality after ICH (13, 25). Although subclinical liver fibrosis or steatosis may not be rare in patients with stroke (26), data are lacking regarding the association between liver fibrosis and HT for patients with AIS. We, therefore, investigated the association between subclinical liver disease, defined using the liver fibrosis score, and HT using a population of patients without overt liver disease. In the present study, we hypothesized that liver fibrosis may be associated with HT in patients with AIS.

## Materials and Methods

### Subjects

We performed a retrospective study using data from the HT database, which collected data on patients admitted to the First Affiliated Hospital of Wenzhou Medical University. All sampled subjects were objectively diagnosed with HT consecutively between October 2011 and March 2019. Approved by the Institutional Review Board and Research Ethics Committee of the First Affiliated Hospital of Wenzhou Medical University, this study was conducted in accordance with the ethical guidelines of the Declaration of Helsinki. No informed consent was required as this study was retrospective and all included data were anonymous.

Patients were sampled if they (1) were 18–90 years old; (2) were hospitalized within 7 days from the onset of stroke; and (3) were identified as AIS after admission by computed tomography (CT) or magnetic resonance imaging (MRI). Subjects were excluded when one of the following conditions was met: (1) a diagnosis of hemorrhagic stroke or transient ischemic attack (TIA); (2) alcohol use; (3) current use of hepatotoxic medications; (4) severe renal disease or known overt liver disease, such as cirrhosis and chronic viral hepatitis B; (5) unavailability of a repeated CT/MRI scan; (6) intravenous thrombolytic therapy received by the patient; or (7) incomplete laboratory data. Ultimately, a total of 185 consecutive participants meeting the requirements were enrolled in this study. According to the same inclusion and exclusion criteria, we included another 199 AIS patients with gender and age matching yet without HT from the stroke unit of our institution.

### Data Collection

A complete survey of all patients was performed using a review of the medical records upon admission. Demographic and clinical data [age, gender, marital status (married or single), and body mass index (BMI)] were documented at baseline. The following stroke risk factors were also identified: hypertension, diabetes mellitus, dyslipidemia, atrial fibrillation, previous history of stroke, current cigarette smoking, and alcohol consumption. Laboratory tests [including white cell count, platelet count, fibrinogen, creatinine, glucose levels (fast blood glucose), HbA1c, aspartate aminotransferase (AST), alanine aminotransferase (ALT), total cholesterol, HDL cholesterol, and LDL cholesterol] and blood pressure measurements were conducted within 24 h after admission. In addition, information on treatment for acute stroke before HT using anticoagulant, antiplatelet, and lipid-lowering agents was collected for all patients. All patients were investigated to clarify the stroke subtype according to the TOAST criteria (27). The size of each infarction area was classified as follows: less than one-half of a lobe was defined as small, and more than one-half of a lobe was defined as large (16, 28, 29). Stroke severity was assessed at admission by experienced neurologists using the National Institutes of Health Stroke Scale (NIHSS) (30). Ranging from 0 to 42, the score on the NIHSS quantifies the extent of neurological deficits.

All patients' platelet count and liver chemistries (AST and ALT) were measured at our hospital's clinical laboratory using routine laboratory methods. All measurements were performed by laboratory personnel blinded to the study samples, baseline characteristics, and outcomes.

### Assessment of Liver Fibrosis

We assessed the extent of liver fibrosis by calculating the non-invasive liver fibrosis score for each subject at the time of admission: the Fibrosis-4 (FIB-4) score. The index is calculated from laboratory data and demographic variables (13, 31, 32) using the following formula: $\left[\left(age\left(years\right)\times AST\left(\frac{U}{L}\right)\right)÷\left(platelet\;count\;(\frac{10^{9}}{L})\times \sqrt{ALT\left(\frac{U}{L}\right)}\right)\right]$. Data on liver imaging are not available in the study. However, this index has been tested to be a non-invasive parameter of liver fibrosis with high diagnostic accuracy in patients with NAFLD (33). The patients were then categorized into two groups according to the optimal cutoff value of the FIB-4 score for further comparisons. The FIB-4 score quantifies the extent of liver fibrosis.

### Definition and Classification of HT

HT was defined as hemorrhage inside the infarct region or parenchyma outside the infarct territory on a follow-up CT scan or MRI (34) which included diffusion-weighted imaging (DWI) and T2-weighted gradient echo, and all patients in this study were detected to have HT within 24 h and 7 days (±2) of the stroke onset. Furthermore, with the aim of diagnosing HT promptly, an imaging examination was performed whenever the patient's clinical condition appeared to worsen. All imaging studies were reviewed retrospectively by consensus of two experienced neurologists blinded to the clinical data, and the presence of HT and its subtypes was confirmed according to the results of CT/MRI tests.

HT can be further categorized radiologically and symptomatically on the basis of the recommendations of the European Cooperative Acute Stroke Study (35). The different types of HT after AIS were divided into hemorrhagic infarction (HI) types 1 and 2 (HI-1 and HI-2) and parenchymal hematoma (PH) types 1 and 2 (PH-1 and PH-2) (35, 36). HT was then further classified as symptomatic or asymptomatic according to whether the neurological deterioration was present. The diagnosis of symptomatic HT (sHT) required an increase of more than 4 points on the NIHSS score, which indicated clinical deterioration; the remaining HT not meeting the requirement was considered asymptomatic HT (asHT) (37).

### Statistical Analysis

Standard statistical methods were used for descriptive statistics. Categorical variables were presented as frequencies and continuous variables as mean ± standard deviation or median (quartiles), as appropriate. Depending on the normality of distribution, the Student *t* test or the Mann–Whitney test was used for continuous variables, as appropriate; the Pearson χ^2^ test or Fisher exact test was used for categorical variables. The receiver operating characteristic (ROC) curve was applied to determine an optimal cutoff value for the FIB-4 score according to the Youden index. Statistical comparisons among the different degrees of HT were conducted using the Kruskal–Wallis test or one-way analysis of variance (ANOVA) with liver fibrosis index as the variable. Known confounding factors and main baseline variables associated with HT identified in the univariate analysis were selected to be covariates. We used multivariate-adjusted binary logistic regression to identify whether the liver fibrosis index might be an independent predictor of HT, HI, and PH. A two-tailed *P*-value of <0.05 was considered to be statistically significant for all tests. All statistical analyses were performed using the Statistical Package for Social Sciences (SPSS 19.0 for Windows, SPSS. Inc., Chicago, IL, USA) and GraphPad Prism, version 8.0.2.

## Results

### Characteristics of the Study Population

**Figure 1:**
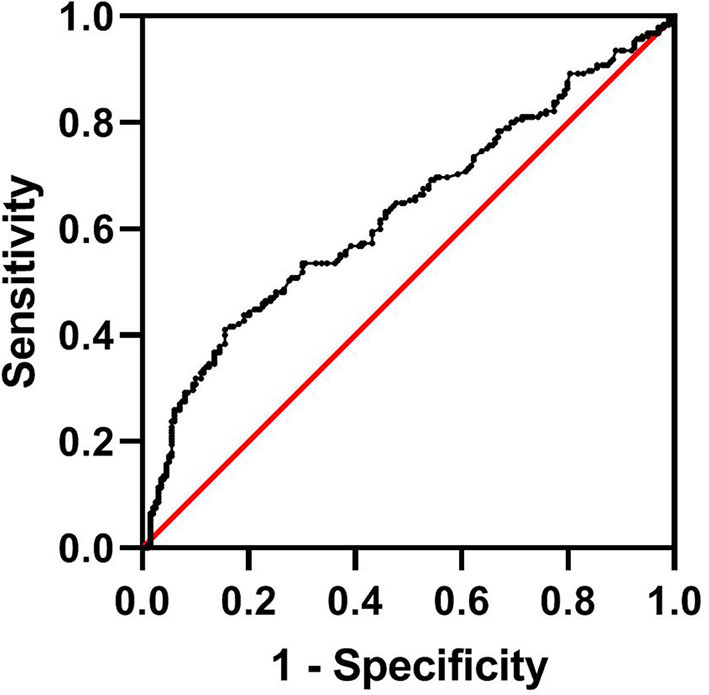
Determination of the cutoff value for the FIB-4 score in AIS patients undergoing HT by ROC analysis. AIS, acute ischemic stroke; HT, hemorrhagic transformation; FIB-4, Fibrosis-4 score; ROC, receiver operating characteristic.

Of the total 287 AIS patients who suffered HT, a total of 102 patients were excluded from the study. These included 4 patients who had known overt liver disease, 93 patients with alcohol use, 3 patients with hepatotoxic medication use, and 2 patients with missing laboratory data. Another 199 age- and sex-matched AIS inpatients without HT from the stroke center at our institution were also enrolled. Therefore, a total of 185 consecutive participants with HT and 199 age- and sex-matched stroke patients without HT were enrolled in the final study. In this study, the median age of patients was 73 years (range 25–96). There were 221 males (57.6%) and 163 females (42.4%). Among the patients with HT, the majority (96.2%) had asHT, while only seven patients (3.8%) were classified as having sHT. According to the imaging features, HI-1 occurred in 31 (17.7%) patients, HI-2 in 63 (36.0%), PH-1 in 40 (22.9%), and PH-2 in 41 (23.4%). The optimal cutoff value for the FIB-4 score determined by the ROC curve was 2.68 (area under the ROC curve: 0.631, 95% CI: 0.575–0.687, *P* < 0.001) with a sensitivity of 41.1% and specificity of 84.4% (Figure 1). The patients were assigned into two groups based on a high (≥2.68) or low (<2.68) FIB-4 score.

The demographic, clinical, and laboratory characteristics of the patients with and without HT are presented in Table 1. As expected, in both groups, patients had generally normal standard liver chemistry examination indices; 7.0% patients without HT and 16.2% with HT had an AST >40 IU/L, while 8.5% patients without HT and 9.2% with HT had an ALT >40 IU/L (Table 1). In this study sample, patients with a history of atrial fibrillation, a large infarction area, and anticoagulation therapy were more likely to undergo HT, while those with a history of antiplatelet or lipid-lowering therapies were less likely to undergo HT. In comparison to patients without HT, those in the HT group had higher baseline white cell count, fibrinogen, glucose levels, AST levels, total cholesterol, and initial NIHSS scores.

**Table 1:** Baseline characteristics of AIS patients with and without HT.

**Variables**	**Non-HT (*n* = 199)**	**HT (*n* = 185)**	***P***
**PATIENT CHARACTERISTICS**			
Age (years)	73.0 (17.0)	73.0 (17.0)	0.891
Male, *n* (%)	115 (57.8%)	106 (57.3%)	0.922
BMI (kg/m^2^)	23.0 (4.0)	23.0 (3.4)	0.898
Married, *n* (%)	183 (92.4%)	173 (93.5%)	0.677
History of stroke, *n* (%)	21 (10.7%)	29 (15.7%)	0.146
History of atrial fibrillation, *n* (%)	20 (10.1%)	79 (42.7%)	0.000^*^
History of hypertension, *n* (%)	145 (73.6%)	121 (65.4%)	0.082
History of diabetes, *n* (%)	59 (29.9%)	44 (23.8%)	0.175
History of dyslipidemia, *n* (%)	14 (7.1%)	12 (6.5%)	0.821
Current smoking, *n* (%)	48 (24.1%)	30 (16.2%)	0.054
**BIOCHEMISTRY AND VITAL SIGNS ON ADMISSION**			
Baseline SBP (mmHg)	158.0 ± 22.7	148.1 ± 24.2	0.000^*^
Baseline DBP (mmHg)	79.5 (18.0)	81.0 (17.0)	0.343
White cell count (×10^9^/L)	6.4 (2.0)	8.3 (3.6)	0.000^*^
Platelets (×10^9^/L)	208.0 (58.0)	191.0 (83.0)	0.006
Fibrinogen (g/L)	3.4 (1.2)	3.9 (1.7)	0.000^*^
Creatinine (μmol/L)	71.5 (28.3)	65.0 (24.0)	0.007
Glucose levels (mmol/L)	5.3 (1.8)	5.7 (2.1)	0.029
HbA1c (%)	6.0 (1.6)	6.0 (1.3)	0.186
AST (units/L)	21.0 (8.0)	26.0 (12.0)	0.000
AST > 40 units/L	14 (7.0%)	30 (16.2%)	0.005
ALT (units/L)	17.0 (11.0)	17.0 (13.0)	0.416
ALT > 40 units/L	17 (8.5%)	17 (9.2%)	0.824
Total cholesterol (mmol/L)	1.9 (2.4)	4.4 (1.6)	0.000^*^
HDL cholesterol (mg/dl)	1.0 (0.3)	1.1 (0.4)	0.113
LDL cholesterol (mmol/L)	2.6 (1.4)	2.6 (1.3)	0.685
Large size of the infarction area, *n* (%)	5 (2.5%)	64 (34.6%)	0.000^*^
NIHSS on admission, median (IQR)	3.0 (4.0)	10.0 (9.0)	0.000^*^
FIB-4	1.92 (1.10)	2.36 (1.77)	0.000^*^
Stroke mechanisms			0.000^*^
Atherosclerotic, *n* (%)	152 (84.9%)	113 (63.1%)	
Cardioembolic, *n* (%)	14 (7.8%)	65 (36.3%)	
Lacunar, *n* (%)	2 (1.1%)	0 (0.0%)	
Other causes, *n* (%)	11 (6.1%)	1 (0.6%)	
**INITIAL TREATMENT IN HOSPITAL**			
Antiplatelets, *n* (%)	180 (90.5%)	104 (56.2%)	0.000^*^
Anticoagulants, *n* (%)	22 (11.1%)	55 (29.7%)	0.000^*^
Lipid-lowering agents, *n* (%)	183 (95.3%)	157 (85.8%)	0.002

* *P < 0.001*.

*AIS, acute ischemic stroke; HT, hemorrhagic transformation; BMI, body mass index; SBP, systolic blood pressure; DBP, diastolic blood pressure; AST, aspartate aminotransferase; ALT, alanine aminotransferase; NIHSS, National Institutes of Health Stroke Scale; FIB-4, Fibrosis-4 score. The bold values were the P values of our object of study*.

The median FIB-4 score was 2.07 (interquartile, 1.30). The baseline demography and disease characteristics of the patients stratified by the FIB-4 score (<2.68 vs. ≥2.68) are shown in Table 2. Compared to patients in the low-FIB-4-score group, those in the high-FIB-4-score group were older and had higher creatinine, total cholesterol, and NIHSS scores and were more likely to undergo atrial fibrillation, a large size of infarction, and anticoagulant treatments. Moreover, the patients in the high-FIB-4-score group tended to have lower platelets and were less likely to undergo diabetes mellitus, antiplatelet, or lipid-lowering therapies in comparison to those in the low-FIB-4-score group. Baseline characteristics of the patients according to the subcategorized groups of HT are shown in Table 3.

**Table 2:** Baseline characteristics of patients with AIS, stratified by FIB-4 scores of <2.68 and ≥2.68.

**Variables**	**FIB-4 score <2.68 (*n* = 277)**	**FIB-4 score ≥ 2.68 (*n* = 107)**	***P***
**PATIENT CHARACTERISTICS**			
Age (years)	70.0 (16.0)	79.0 (8.0)	0.000^*^
Male, *n* (%)	159 (57.4%)	62 (57.9%)	0.923
BMI (kg/m^2^)	23.4 ± 3.1	21.3 ± 2.4	0.006
History of atrial fibrillation, *n* (%)	54 (19.6%)	45 (42.1%)	0.000^*^
History of hypertension, *n* (%)	192 (69.8%)	74 (69.2%)	0.900
History of diabetes mellitus, *n* (%)	87 (31.6%)	16 (15.0%)	0.001
History of dyslipidemia, *n* (%)	20 (7.3%)	6 (5.6%)	0.556
Current smoking, *n* (%)	68 (24.5%)	10 (9.3%)	0.001
**BIOCHEMISTRY AND VITAL SIGNS ON ADMISSION**			
Baseline SBP (mmHg)	152.8 ± 25.0	154.1 ± 20.8	0.621
White cell count (×10^9^/L)	7.0 (2.8)	7.5 (3.7)	0.266
Platelets (×10^9^/L)	215.0 (65.0)	162.0 (66.0)	0.000^*^
Fibrinogen (g/L)	3.6 (1.3)	3.7 (1.6)	0.315
Creatinine (μmol/L)	68.0 (27.8)	72.0 (28.0)	0.083
Glucose levels (mmol/L)	5.5 (2.2)	5.6 (1.7)	0.935
AST (units/L)	21.0 (9.5)	29.0 (18.0)	0.000^*^
AST > 40 units/L	14 (5.1%)	30 (28.0%)	0.000^*^
ALT (units/L)	17.0 (11.0)	16.0 (12.0)	0.268
ALT > 40 units/L	22 (7.9%)	12 (11.2%)	0.311
Total cholesterol (mmol/L)	3.2 (3.2)	4.0 (1.6)	0.001
HDL cholesterol (mg/dl)	1.0 (0.3)	1.1 (0.3)	0.024
LDL cholesterol (mmol/L)	2.6 (1.4)	2.5 (1.2)	0.099
Large size of the infarction area, *n* (%)	35 (12.7%)	34 (31.8%)	0.000^*^
NIHSS on admission, median (IQR)	4.0 (6.0)	10.0 (10.0)	0.000^*^
HT, *n* (%)	109 (39.4%)	76 (71.0%)	0.000^*^
Stroke mechanisms			0.000^*^
Atherosclerotic, *n* (%)	201 (78.8%)	64 (62.1%)	
Cardioembolic, *n* (%)	41 (16.1%)	38 (36.9%)	
Lacunar, *n* (%)	1 (0.4%)	1 (1.0%)	
Other causes, *n* (%)	12 (4.7%)	0(0.0%)	
**INITIAL TREATMENT IN HOSPITAL**			
Antiplatelets, *n* (%)	219 (79.1%)	65 (60.7%)	0.000^*^
Anticoagulants, *n* (%)	41 (14.8%)	36 (33.6%)	0.000^*^
Lipid-lowering agents, *n* (%)	254 (93.7%)	86 (82.7%)	0.001

* *P < 0.001*.

*AIS, acute ischemic stroke; HT, hemorrhagic transformation; BMI, body mass index; SBP, systolic blood pressure; AST, aspartate aminotransferase; ALT, alanine aminotransferase; NIHSS, National Institutes of Health Stroke Scale; FIB-4, Fibrosis-4 score. The bold values were the P values of our object of study*.

**Table 3:** Baseline characteristics of the patients according to the subcategorized groups of HT.

**Variables**	**Non-HT (*n* = 199)**	**HI (*n* = 94)**	**PH (*n* = 81)**	***P***
**PATIENT CHARACTERISTICS**				
Age (years)	73.0 (17.0)	73.0 (16.0)	71.0 (22.0)	0.501
Male, *n* (%)	115 (57.8%)	53.0 (56.4%)	46 (56.8%)	0.971
BMI (kg/m^2^)	23.0 (4.0)	22.8 (3.7)	23.8 (4.7)	0.904
History of atrial fibrillation, *n* (%)	20 (10.1%)	34 (36.2%)	43 (53.1%)	0.000^*^
History of hypertension, *n* (%)	145 (73.6%)	65 (69.1%)	49 (60.5%)	0.096
History of diabetes mellitus, *n* (%)	59 (29.9%)	22 (23.4%)	19 (23.5%)	0.367
History of dyslipidemia, *n* (%)	14 (7.1%)	9 (9.6%)	3 (3.8%)	0.324
Current smoking, *n* (%)	48 (24.1%)	15 (16.0%)	13 (16.0%)	0.150
**BIOCHEMISTRY AND VITAL SIGNS ON ADMISSION**				
Baseline SBP (mmHg)	158.0 ± 22.7	149.7 ± 25.3	145.0 ± 22.6	0.000^*^
White cell count (×10^9^/L)	6.4 (2.0)	7.6 (3.8)	8.6 (3.3)	0.000^*^
Platelets (×10^9^/L)	208.0 (58.0)	196.0 (65.8)	183.0 (98.0)	0.016
Fibrinogen (g/L)	3.4 (1.2)	3.9 (1.5)	3.9 (2.0)	0.000^*^
Creatinine (μmol/L)	71.5 (28.3)	64.0 (22.5)	69.0 (23.0)	0.007
Glucose levels (mmol/L)	5.3 (1.8)	5.7 (2.1)	5.8 (2.1)	0.026
AST (units/L)	21.0 (8.0)	26.5 (11.3)	24.0 (12.5)	0.000^*^
AST > 40 units/L	14 (7.0%)	16 (17.0%)	11 (13.6%)	0.027
ALT (units/L)	17.0 (11.0)	17.5 (13.3)	16.0 (12.5)	0.283
ALT > 40 units/L	17 (8.5%)	7 (7.4%)	8 (9.9%)	0.849
FIB-4	1.92 (1.10)	2.29 (1.70)	2.36 (1.76)	0.000^*^
Total cholesterol (mmol/L)	1.9 (2.4)	4.3 (2.0)	4.5 (1.3)	0.000^*^
HDL cholesterol (mg/dl)	1.0 (0.3)	1.1 (0.3)	1.1 (0.4)	0.073
LDL cholesterol (mmol/L)	2.6 (1.4)	2.5 (1.4)	2.7 (1.2)	0.793
Large size of the infarction area, *n* (%)	5 (2.5%)	33 (35.1%)	30 (37.0%)	0.000^*^
NIHSS on admission, median (IQR)	3.0 (4.0)	8.0 (9.0)	12.0 (9.0)	0.000^*^
Stroke mechanisms				0.000^*^
Atherosclerotic, *n* (%)	152 (84.9%)	68 (76.4%)	38 (47.5%)	
Cardioembolic, *n* (%)	14 (7.8%)	21 (23.6%)	41 (51.2%)	
Lacunar, *n* (%)	2 (1.1%)	0 (0.0%)	0 (0.0%)	
Other causes, *n* (%)	11 (6.1%)	0 (0.0%)	1 (1.3%)	
**INITIAL TREATMENT IN HOSPITAL**				
Antiplatelets, *n* (%)	180 (90.5%)	54 (57.4%)	44 (54.3%)	0.000^*^
Anticoagulants, *n* (%)	22 (11.1%)	25 (26.6%)	28 (34.6%)	0.000^*^
Lipid-lowering agents, *n* (%)	183 (95.3%)	80 (87.0%)	67 (82.7%)	0.002

* *P < 0.001*.

*HT, hemorrhagic transformation; HI, hemorrhagic infarct; PH, parenchymal hematoma; BMI, body mass index; SBP, systolic blood pressure; AST, aspartate aminotransferase; ALT, alanine aminotransferase; NIHSS, National Institutes of Health Stroke Scale; FIB-4, Fibrosis-4 score. The bold values were the P values of our object of study*.

### Fibrosis Indices and HT

**Figure 2:**
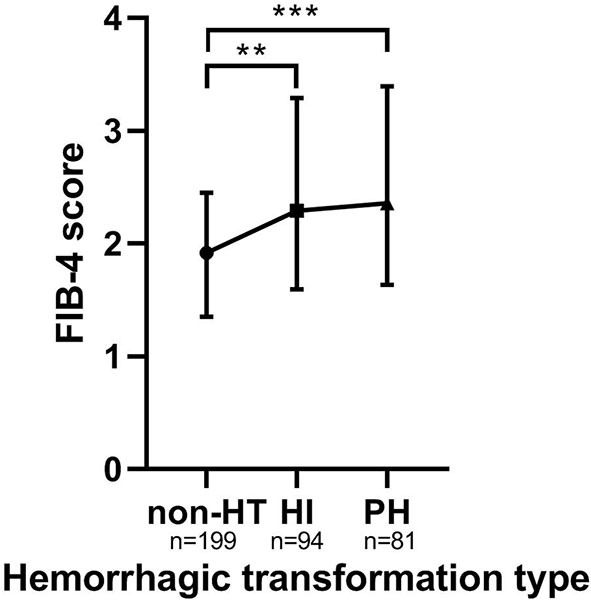
The FIB-4 score in the subcategorized groups of HT. Each data point and error bar corresponds to the median and interquartile range of the FIB-4 score by the subcategorized groups of HT. HT, hemorrhagic transformation; HI, hemorrhagic infarct; PH, parenchymal hematoma. **P < 0.01; ***P < 0.001.

**Figure 3:**
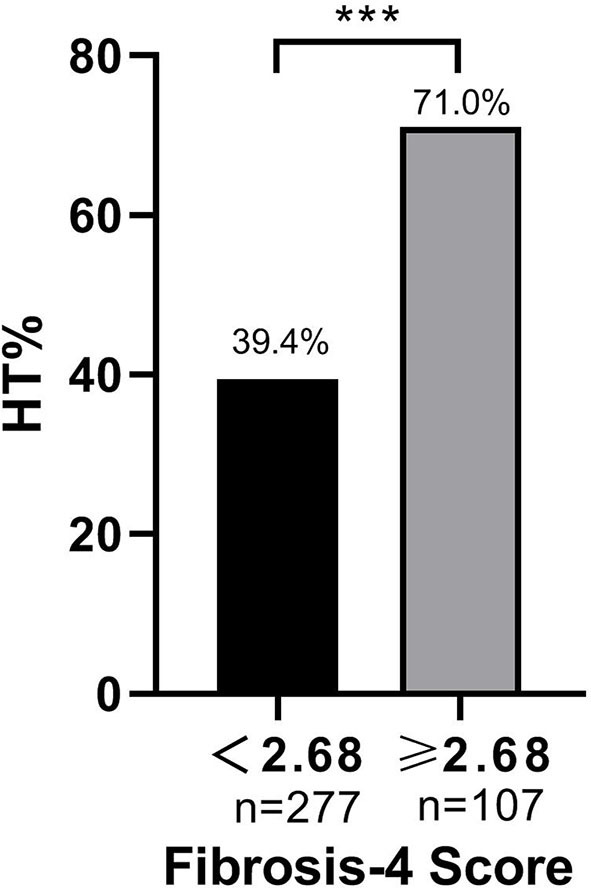
The incidence of HT at different FIB-4 score. ***P < 0.001.

Baseline FIB-4 scores were significantly higher in patients with HT than in those without HT (1.92 ± 1.10 vs. 2.36 ± 1.77, *P* < 0.001; Table 1). As for the radiological status of HT, baseline FIB-4 scores were significantly different among subjects in three groups (*H* = 18.924, *P* < 0.001 by Kruskal–Wallis test; Table 3, Figure 2). Additionally, Figure 2 shows that the FIB-4 scores were significantly higher in patients with HI or PH when compared to those without HT after the Bonferroni modification [2.29 (1.60–3.29) vs. 1.92 (1.35–2.45), *P* = 0.002; 2.36 (1.64–3.40) vs. 1.92 (1.35–2.45), *P* < 0.001, respectively]. However, there is no significant difference in FIB-4 scores between patients with HI and PH. The proportions of subjects diagnosed with HT were higher in the high-FIB-4-score group than in the low-FIB-4-score group (39.4 vs. 71.0%, chi-square test *P* < 0.001; Figure 3).

**Figure 4:**
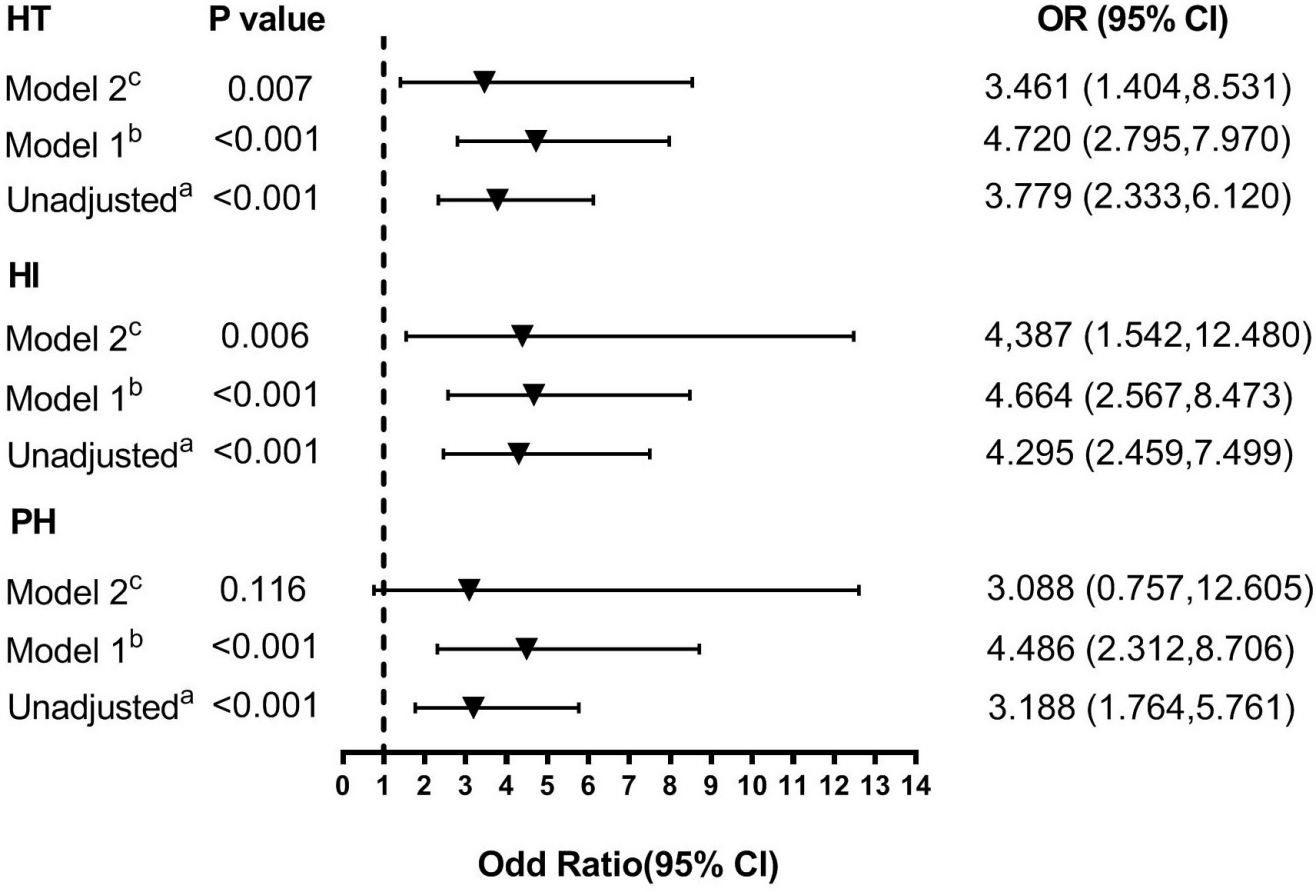
Multivariate adjusted odds ratios for the association between FIB-4 score and the subcategorized groups of HT (including HT, HI, and PH, respectively). OR, odds radio; CI, confidence level; HI, hemorrhagic infarct; HT: hemorrhagic transformation; PH, parenchymal hematoma. **(A)** Reference OR (1.000) is an FIB-4 score <2.68. **(B)** Model 1: adjusted for age and sex. **(C)** Model 2: adjusted for covariates from Model 1 and further adjusted for vascular risk factors (history of hypertension, atrial fibrillation, diabetes, dyslipidemia, and current smoking) and systolic blood pressure, stroke mechanism, size of the infarction area, baseline NIHSS score, baseline white cell counts, fibrinogen, total cholesterol, and anticoagulant, antiplatelet, and lipid-lowering therapies.

In univariate analyses, patients in the high-FIB-4-score group were associated with an increased risk for HT with an odds ratio (OR) (95% CI = 2.333–6.120, *P* < 0.001) of 3.779 without adjustment and showed significant associations with a high risk of HI (OR = 4.295, 95% CI = 2.459–7.499, *P* < 0.001) as well as PH (OR = 3.188, 95% CI = 1.764–5.761, *P* < 0.001; Figure 4). Risk for HT was also linked with atrial fibrillation, elevated baseline NIHSS score, total cholesterol, and lack of antiplatelet use.

A distinction was made between the two adjusted multivariable models, and the covariates for each model were the same for each dependent variable (Figure 4). Compared with the patients in the low-FIB-4-score group, those in the high-FIB-4-score group had an OR (95% CI) of 4.720 (2.795, 7.970) for HT, 4.664 (2.567, 8.473) for HI, and 4.486 (2.312, 8.706) for PH after adjusting for age and gender (all *P* < 0.001; Figure 4). After further adjusting for factors already identified as risk factors for HT and those potential factors detected in the univariate analysis (Model 2: adjusting for age, gender, SBP, baseline NIHSS score, hypertension, atrial fibrillation, diabetes, dyslipidemia, smoking status, stroke mechanism, size of the infarction area, baseline white cell count, fibrinogen, total cholesterol, and anticoagulant, antiplatelet, and lipid-lowering therapies), the risk of HT (OR 3.461 [95% CI, 1.404–8.531]) and HI (OR 4.387 [95% CI, 1.542–12.480]) remained significant (*P* = 0.007 and *P* = 0.006, respectively). However, the risk of PH was not significantly higher in the high-FIB-4-score group (OR 3.088 [95% CI, 0.757–12.605], *P* = 0.116).

## Discussion

In this study, the liver fibrosis index was independently associated with an increased risk for HT, even after adjustment for potential and known confounders. To our knowledge, this is the first study to explore and analyze the relationship between liver fibrosis and HT in patients with AIS. It is noteworthy that these associations were found in a study population in which standard liver enzyme levels were commonly normal.

The liver dysfunction has been found to contribute to hematoma expansion in spontaneous ICH (38–41) and HT in AIS (42). In addition, the HAS-BLED score, which includes liver dysfunction as a fundamental item, is widely used for evaluating hemorrhagic risk (43). One such study found that derangements in individual liver enzymes, such as increased serum alkaline phosphatase, were associated with high-risk sHT in AIS patients (44). However, these studies were mostly based on individual, non-specific hepatic enzyme tests in populations with heavy alcohol use. In contrast, our study, in accordance with prior studies, suggested a novel association between a relatively validated liver fibrosis index and HT in patients with AIS. Several studies have identified that standard hepatic chemistries are generally normal in patients with chronic liver disease. Moreover, the proportion of individuals with imaging evidence of significant liver fibrosis whose levels of transaminase are normal is nearly three quarters (9, 45). Interestingly, a recent retrospective cohort study showed the associations between liver fibrosis indices and admission hematoma volume, hematoma enlargement, and 3-month mortality despite largely normal standard hepatic chemistries among patients with ICH (13). These findings inspired us to consider the presence of subclinical liver fibrosis and raised the possibility that liver fibrosis is associated with HT in AIS patients without clinically overt liver disease.

Recently, some reports have considered either patients without known overt liver disease as well as alcohol use or isolated derangements in individual hepatic enzyme levels when analyzing the association between liver disease and outcomes of stroke. Tan et al. found that in univariable analysis, subclinical abnormalities in individual liver enzymes were linked with poor prognosis in ICH, but these associations were not significant after adjusting for confounding factors (46). In another study, associations were found between liver cirrhosis and higher in-hospital mortality in ICH among patients with overt liver disease caused by virus or alcohol (47). Additionally, with regard to alcohol use or clinically overt liver disease, recent studies have suggested that liver fibrosis was independently associated with the risk of incident of cardiovascular events and prognosis of stroke (3, 5, 48). Based upon these data, our study demonstrated an association between the liver fibrosis score and HT among AIS patients with largely normal standard liver chemistries. sHT is an important clinical outcome of AIS patients, increasing the worse prognosis together with asHT (49). According to a recent study, about 8% of patients will develop sHT and subsequent worsening of the outcomes after thrombolysis therapies (50). However, due to the limited number of patients with sHT in our study, we did not perform this analysis on the association between liver fibrosis and sHT. More studies need to be conducted to further analyze the relationship between liver fibrosis and sHT. In the process of research, we have hypothesized that the liver fibrosis index was a predictor of HT severity. It was previously shown that PH represented poor clinical outcomes in stroke patients. However, there was no statistically significant difference between HI and PH in this study. We hope that more research can be designed to further confirm the results.

Although the mechanisms underlying the association between liver fibrosis and HT remain obscure, several explanations may account for the observed association. First, it has been demonstrated that liver fibrosis increases endothelial dysfunction (51, 52). Second, inflammation is typically present in all liver disease stages and associated with the development of liver fibrosis (53). An inflammatory response resulting in the increasing release of plasma biomarkers of inflammation can aggravate endothelial dysfunction (8, 54). Third, according to previous study, oxidative stress represents a shared pathophysiological disorder between liver disease and cardiovascular risk factors (55). Studies have suggested that oxidative stress could act as a potential trigger of HT by undermining the integrity of both basal lamina and endothelial tight junctions in the blood–brain barrier (56). Furthermore, liver fibrosis may contribute to HT through the mechanism of subclinical coagulopathy (57). Progression of liver fibrosis reduces the production of thrombopoietin by hepatocytes and, hence, reduced platelet production (58). Finally, fibrogenesis can be intensified by interfering with the fibrolytic activity of the TIMP-1/MMP system, which is closely related to HT in patients with AIS (59, 60). Though unconfirmed, the expression of the MMP family in liver fibrosis may play a vital role in HT.

There are limitations in our study. First, patients with thrombolytic therapy were excluded in this study, and a subgroup analysis of patients with and without thrombolytic therapy was not performed. Therefore, we expect further investigation to eliminate the effect of thrombolytic therapy on the results in this study. Second, we could not establish causality as this study is a retrospective, single-center study. Further prospective, multicenter studies are still needed to confirm the results. Third, we purposefully excluded the patients who consumed alcohol because the liver fibrosis index was proven to be accurate in patients with NAFLD. Fourth, the infarct size was taken as a categorical variable in the analysis, and it is better to calculate the infarct size using the Alberta Stroke Program Early CT Score (ASPECTS) system by trained radiologists. Moreover, we did not analyze the relationship between liver fibrosis and sHT due to the limited sample size of this study and because the majority of patients with HT were asymptomatic. Finally, we did not have liver imaging or liver biopsy data to ascertain the presence of liver fibrosis in our study population though two simple, non-invasive, and validated biomarkers were used to assess the extent of liver fibrosis.

## Conclusion

In conclusion, our study demonstrated that liver fibrosis was associated with HT among patients with AIS despite largely normal standard liver chemistries.

## Data Availability Statement

The data analyzed in this study is subject to the following licenses/restrictions: Research data are not shared. Requests to access these datasets should be directed to Jin-Cai He, hjc@wmu.edu.cn.

## Ethics Statement

The studies including human participants were reviewed and approved by the Institutional Review Board and Research Ethics Committee of the First Affiliated Hospital of Wenzhou Medical University. Written informed consent was not required as the study was a retrospective study.

## Author Contributions

C-XY and J-CH designed the study. Y-TR and Y-YZ interpreted data. C-XY wrote the manuscript. H-RC, Q-QC, and Y-BC prepared the figures. W-LH and Y-TR did the statistical analyses. G-QH, Q-QC, and Y-YZ screened and extracted the data. J-CH supervised the study. All authors have made an intellectual contribution to the manuscript and approved the submission. All authors contributed to the article and approved the submitted version.

## Conflict of Interest

The authors declare that the research was conducted in the absence of any commercial or financial relationships that could be construed as a potential conflict of interest.
